# The impact of transcranial direct current stimulation on inhibitory control in young adults

**DOI:** 10.1002/brb3.332

**Published:** 2015-03-19

**Authors:** Andrea M Loftus, Ozgur Yalcin, Frank D Baughman, Eric J Vanman, Martin S Hagger

**Affiliations:** 1 Curtin Neuroscience Laboratory, School of Psychology and Speech Pathology, Curtin UniversityPerth, Western Australia, Australia; 2 School of Psychology, University of QueenslandBrisbane, Queensland, Australia; 3 Health Psychology and Behavioural Medicine Group and Laboratory of Self-Regulation (LaSeR), School of Psychology and Speech Pathology, Curtin UniversityPerth, Western Australia, Australia

**Keywords:** Dorsolateral prefrontal cortex, executive functioning, self-control, stroop, transcranial direct current stimulation

## Abstract

**Background:**

There is increasing evidence that the dorso-lateral prefrontal cortex (DLPFC), a brain region related to reward and motivational processes, is involved in effective response inhibition and that decreased activity in this region coincides with reduced inhibitory capacity. Using transcranial direct current stimulation (tDCS) to manipulate cortical activation, this study examined whether cross-hemispheric tDCS over the DLPFC affected performance on an inhibitory control task.

**Methods:**

Neurologically intact participants performed a modified Stroop color-word matching task before and after completing one of two tDCS conditions; (1) anodal stimulation over the left DLPFC or (2) sham tDCS.

**Results:**

There was a statistically significant effect of tDCS condition on Stroop reaction time (RT) pre-post tDCS change scores. Participants who received anodal stimulation over the left DLPFC demonstrated statistically significant faster RT change scores on the Stroop items compared to participants in the sham condition. Although errors on Stroop incongruent items decreased before and after receiving the tDCS treatment, there were no significant differences in errors on Stroop items between the anodal stimulation over left DLPFC and sham tDCS conditions. Anodal tDCS, which is known to elevate neural excitation, may have enhanced activation levels in the left DLPFC and minimized impairment of inhibitory control, resulting in better task performance.

**Conclusions:**

Current findings provide preliminary evidence that increased excitation of the left DLPFC improves inhibitory control and are a step toward understanding the potential of tDCS for moderating deficits in inhibitory control.

## Introduction

Recent research indicates that inhibitory control, or response inhibition, is a key feature of self-control and may impact upon an individual's ability to inhibit impulsive responses to stimuli (Friese et al. 2008; Hofmann et al. 2009; Fujita 2011). For example, a compromised ability to inhibit impulsive responses is associated with increased consumption of high calorie food (Guerrieri et al. 2012), higher alcohol intake (Houben et al. 2011), and a propensity toward obesity (Guerrieri et al. 2008). Furthermore, research has demonstrated that impaired inhibitory control is significantly related to many behaviors that require impulse suppression such as food consumption (Hagger et al. 2013b), smoking urges (Hagger et al. 2013a), and alcohol-seeking behavior (Muraven and Shmueli 2006).

A number of studies suggest that modulating an individual's inhibitory control for alcohol and food-related cues impacts upon their subsequent consumption of alcohol and palatable foods in ostensible taste-and-rate tasks (Houben et al. 2011; Jones et al. 2011). For example, Houben et al. (2011) examined whether increasing or decreasing inhibitory control impacted food intake. Participants completed an initial response inhibition task, the stop-signal task (SST; Logan et al. 1997). Following this, participants completed either (1) an inhibition condition in which one type of food was always paired with a stop signal, or (2) an impulsive condition in which another type of food was never paired with a stop signal. The consistent mapping of one type of food onto stop signals was purported to increase inhibitory control capacity for that food type. Conversely, the type of food which was never paired with a stop signal (impulsive condition) would lead to decreased ability to inhibit responses to those stimuli and increase impulsivity in response to that food. Participants also completed a bogus taste test of the foods presented in the SST, during which calorific consumption was monitored. Increasing inhibition toward a particular food decreased the subsequent consumption of that food in the taste-test phase of the study, whereas decreasing inhibition (increasing impulsivity) toward a particular food increased intake of that food.

In a similar study, Jones et al. (2011) examined the impact of priming inhibitory control using an SST paradigm on alcohol-seeking behavior. Participants were randomly assigned to SST groups that differed in terms of the emphasis placed upon the importance of successful inhibition. Participants in the ‘disinhibition’ group were informed that rapid responding was the most important task, whereas participants in the ‘restraint’ group were informed that successful inhibition was to be prioritized. Participants were then asked to rate the pleasantness of different drinks, including a beer they believed to contain alcohol. The results indicated that participants in the ‘disinhibition’ group, who had been informed to prioritize response speed, consumed more beer than participants in the ‘restraint’ group who were instructed to prioritise inhibition. These findings led Jones et al. to suggest that a temporary loss of inhibitory control impacts upon motivated behavior such as alcohol-seeking.

These studies provide evidence that ‘strengthening’ or ‘training’ inhibitory control through repeated ‘practice’ on response inhibition tasks impacts upon behavior in situations requiring self-control, potentially by increasing resistance to temptation and impulsive cue-driven responding. These findings have been replicated elsewhere (e.g., Veling et al. 2011, 2013a,b; Todd and Mullan 2013). The underlying neural mechanism for such strengthening of inhibitory control, however, remains unclear. A number of studies implicate dorso-lateral prefrontal cortex (DLPFC) activation during tasks and behaviors involving response inhibition and self-control (MacDonald et al. 2000; Knoch et al. 2006; Glascher et al. 2009; Hare et al. 2009; Figner et al. 2010; Heatherton and Wagner 2011; Friese et al. 2013). For example, Steinbeis et al. (2012) examined children's decision-making abilities while playing two different games, only one of which required participants to exert self-control. Functional magnetic resonance imaging (fMRI) results revealed left DLPFC activation *only* when participants played the game which required them to exert self-control. Similarly, Friese et al. (2013) demonstrated that engaging in an emotion suppression task requiring individuals to actively inhibit their responses to emotionally evocative stimuli coincided with reduced performance on a subsequent Stoop color-word matching task, a task that has frequently been implicated in the literature as a measure of response inhibition and the inhibition component of self-control.^1^ Importantly, the decrement in performance coincided with reduced activity in the DLPFC verified by fMRI. Deficits in this brain region, shown to be correlated with reward and motivational processes, appear to be implicated in effective response inhibition and decreased activity in this region coincides with reduced capacity for response inhibition (Heatherton 2011; Hedgcock et al. 2012; Friese et al. 2013; Hagger and Chatzisarantis 2013). Given this evidence, we propose that increased activity in the DLPFC may enhance inhibitory control and, therefore, performance on tasks requiring self-control. In this study, we stimulated DLPFC activity using transcranial direct current stimulation (tDCS) and examined subsequent effects on performance on an inhibitory control task.

tDCS is a noninvasive method of brain stimulation that can be used to modulate cortical excitability. When applied to the skull, tDCS penetrates the underlying cortex and increases (anodal) or decreases (cathodal) cortical excitability in that area (Nitsche and Paulus 2000; Zaghi et al. 2010; Lang et al. 2011). tDCS can be applied in a cross-hemispheric manner, whereby the anodal electrode is applied to one hemisphere and the cathodal to the other. Recent research suggests that cross-hemispheric tDCS over DLPFC improves performance on numerous tasks associated with executive functioning including task-switching tasks (Leite et al. 2012) and working memory (Jeon and Han 2012), and as well as modifying impulsive responses such as amelioration of risk-taking behaviors (Fecteau et al. 2007; Boggio et al. 2010). For example, Fecteau et al. (2007) examined the impact of cross-hemispheric tDCS over DLPFC on the Balloon Analogue Risk Task (BART), a behavioral analog of risk-taking and impulsivity that correlates well with ‘real world’ measures of risk-related behaviors (Lejuez et al. 2002). Participants received either: (1) anodal over the right and cathodal over the left DLPFC (ARCL), (2) anodal over the left and cathodal over the right DLPFC (ALCR), or (3) sham stimulation during the risk task. Participants in the ARCL group demonstrated less risk taking on the BART compared to those in sham or ALCR tDCS groups. This led Fecteau et al. (2007) to suggest that the interhemispheric balance of activation across the DLPFC cortices contributes to decision-making behavior, and that altering this balance impacts upon risk taking.

This study examined the impact of cross-hemispheric tDCS over the DLPFC on inhibitory control using a Stroop color-word matching task in neurologically intact participants. The techniques associated with tDCS are a developing science and precise predictions are somewhat difficult to make, since effective dose and duration for a given task remain unclear (Jacobson et al. 2012). Based on the recent finding that left DLPFC is primarily activated during response inhibition and self-control tasks, we predicted that participants receiving an anodal tDCS over the left DLPFC, the configuration linked with increased activity in the DLPFC and performance on tasks correlated with processing in this region, would be more likely to improve inhibitory control than participants receiving sham tDCS. Specifically, we predict that participants will demonstrate better Stroop-task performance, as indicated by faster averaged response latency and decreased error rates, after receiving anodal tDCS over the left DLPFC and controlling for baseline, relative to Stroop performance of participants receiving the sham tDCS.

## Method

### Participants

Twenty-eight neurologically intact, right-handed (in accord with the Edinburgh Handedness Inventory; Oldfield 1971) undergraduate students took part in the study (18 females, mean age 24.5 years). All participants had normal or corrected to normal vision. The study was approved by the Curtin University Human Research Ethics Committee. All participants provided written informed consent.

### Procedure and materials

Participants were randomly assigned to one of two tDCS conditions; (1) anodal stimulation of left dorso-lateral prefrontal cortex (DLPFC) or (2) sham (no tDCS). Participants were naive to the tDCS condition to which they were assigned. Within each session, participants completed the following tasks in this order: (1) pre-tDCS Stroop task, (2) tDCS, and (3) post-tDCS Stroop task. Fourteen participants were assigned to each tDCS condition.

#### Stroop task

A computerized version of a modified Stroop color-word matching task was used to measure inhibitory control. Following the task instructions, two strings of letters, presented in 140 point font size, were simultaneously presented on a LCD computer screen, one at the top and one at the bottom, 100 pixels either side of the midpoint of the screen. The string of letters at the top of the screen was always presented in one of four colors (yellow, red, blue, or green). These letters were either nonwords (i.e., a set of randomly scrambled letters) or spelled a color word (i.e., yellow, red, blue, or green). The string of letters at the bottom of the screen was always presented in gray color. Participants were instructed to decide, as quickly and accurately as possible, whether the color of the letter string at the top of the screen matched the meaning (name of color) of the string at the bottom. Participants responded by clicking the left mouse button for a ‘match’ response and the right mouse button for a ‘non-match’ response. Participants first completed seven practice trials, followed by 64 test trials (32 neutral and 32 incongruent). In the neutral trials, the target word was a random string of letters (e.g., “NSGL”) in which the color of the string presented at the top of the screen matched the meaning of the string presented at the bottom of the screen. In incongruent trials, the letter string presented at the top of the screen was a real word that differed in both color and meaning (e.g., the word “BLUE” in the color red) to the string presented at the bottom of the screen. Trials were counterbalanced and randomly presented. Stimuli remained on the screen until a response was given, or until 5 sec had passed. A blank screen was then briefly presented for 1000 ms. The presentation of a fixation cross of size 100 × 100 pixels in the center of the screen indicated the start of a new trial. The task took approximately 10 min to complete. Reaction time (RT) to each stimulus item was recorded in millisecond (ms) from the onset of the stimulus presentation until the response was detected of 5 sec had passed. Errors were recorded as the number of incorrect trials.^2^

#### Transcranial direct current stimulation (tDCS)

Immediately following the pre-tDCS Stroop task, each participant commenced the tDCS phase of the study. tDCS was delivered by a battery-driven, constant current stimulator (Soterix™ 1x1). A constant current of 2 mA was applied for 10 min with a pair of 35 cm^2^ sponge electrodes soaked in saline solution (equivalent to 0.057 mA/cm^2^). There was a ramp up/ramp down period of 30 sec at the start and end of tDCS. Participants received either anodal stimulation over the left DLPFC or sham tDCS. Anodal stimulation over the left DLPFC was performed with the anode placed over F3 (using the 10–20 system) and the cathode placed over the right DLPFC (F4 using the 10–20 system). Sham stimulation was conducted with the same montage, with 30 sec of tDCS applied at onset, after which the current stimulator was de-ramped. tDCS was administered for 10 min, during which time the participant watched a short video (comedy sketch, all participants watched same video). Following administration of tDCS, participants completed the post-tDCS Stroop task, which was the same as the pre-tDCS Stroop task except for the order of trials. Upon completion of tDCS, all participants were asked if they could tell whether they received stimulation or not (yes or no response).

## Results

All participants successfully completed the experiment and there were no missing data. No participant reported any adverse effects of tDCS. Data from all 28 participants were used in analysis.

### The stroop effect

**Figure 1:**
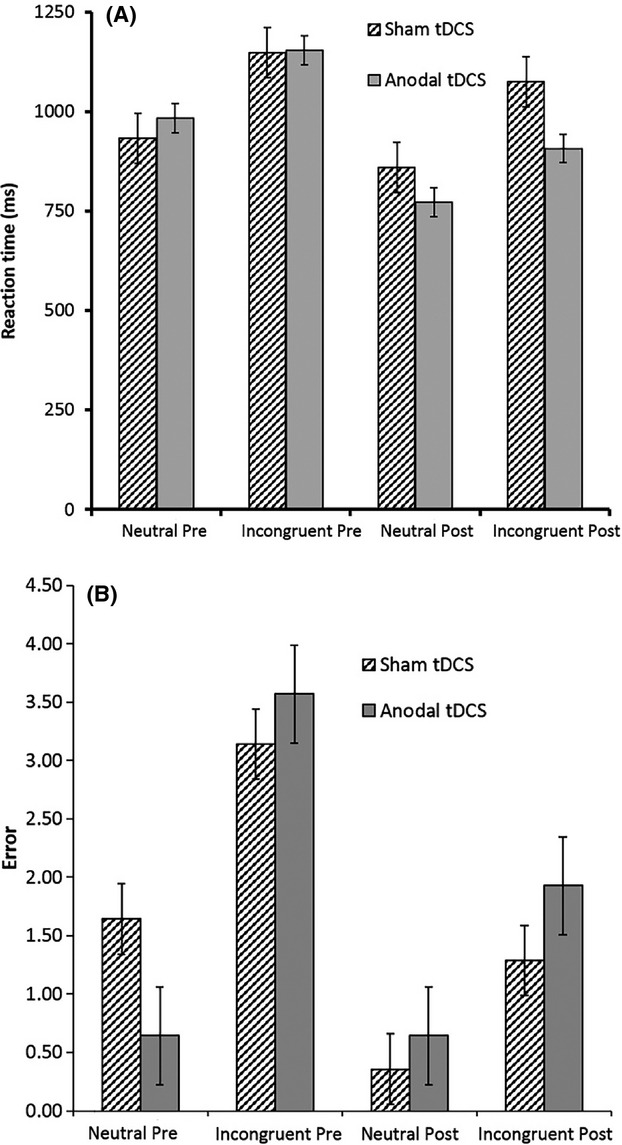
(A) Mean reaction times for each trial type (neutral, incongruent) at each time (pre-tDCS, post-tDCS), including standard error. (B) Mean error scores for each trial type (neutral, incongruent) at each time (pre-tDCS, post-tDCS), including standard error.

Bonferroni-adjusted paired-samples *t*-tests revealed statistically significant differences between neutral and incongruent Stroop stimuli words pre-tDCS for reaction time (RT), *t*_27_ = −8.14, *P *< 0.001, *d* = 1.11, and error rates, *t*_27_ = −4.61, *P *< 0.001, *d* = 0.97. Participants responded faster (M = 957.82, SD* *= 147.69) and exhibited fewer errors (M = 1.14, SD* *= 1.60) on neutral trials compared to incongruent trials (RT: M* *= 1150.37 SD* *= 198.61; Errors: M* *= 3.36, SD* *= 2.25). Analogously, participants also exhibited statistically significant faster RTs (M = 815.46, SD* *= 185.33; *t*_27_ = −5.35, *P *< 0.001, *d* = 0.79) with fewer errors (M = 0.50, SD* *= 0.79; *t*_27_ = −3.39 *P *= 0.002, *d *= 0.89) on post-tDCS neutral trials compared to incongruent trials (RT: M* *= 990.44, SD* *= 250.68; Errors: M* *= 1.60, SD* *= 1.57). These findings are consistent with the Stroop effect in that participants are expected to respond more quickly and accurately on neutral trials compared to incongruent trials. Mean actual RTs and error scores for each trial type and tDCS condition are presented for both times in Figures1A and B.

### Reaction time

Reaction time scores were entered into analysis as raw scores. To examine the impact of tDCS group on neutral and incongruent trials of the Stroop task, a repeated measures analysis of variance (ANOVA) with tDCS condition (anodal, sham) as the between-groups factor and trial type (neutral, incongruent) and time (pre-tDCS, post-tDCS) as within-groups factors was used. There was no statistically significant main effect of tDCS condition, *F*_1,26_ = 0.591, *P *> 0.05, partial *η*^2^ = 0.02. There were statistically significant main effects of time, *F*_1,26_ = 38.16, *P *< 0.001, partial *η*^2^ = 0.60, and trial type, *F*_1,26_ = 51.75, *P *< 0.001, partial *η*^2^ = 0.66. There was a statistically significant tDCS condition x time interaction effect, *F*_1,26_ = 10.08, *P *< 0.05, partial *η*^2^ = 0.25. There were no other statistically significant interaction effects.

To facilitate analysis of the tDCS condition × time interaction effect, RT change scores were calculated by subtracting the mean pre-tDCS RT from the mean post-tDCS RT for each participant, with negative RT change scores indicative of reduced pre-post RT (i.e., the participant was faster) and positive RT change scores indicative of increased pre-post RT (i.e., the participant was slower). Adjusted independent samples *t*-tests revealed a statistically significant difference between sham and ALCR tDCS conditions on RT change scores for neutral trials, *t*_28_ = −3.07, *P *< 0.05, *d *= 1.16, and incongruent trials, *t*_28_ = −2.74, *P *< 0.05, *d *= 1.04. For neutral trials, those who received anodal tDCS demonstrated greater reduction in RT (M* *= −210.70, SD* *= 107.64) compared to those in the sham condition (M* *= −74.03, SD* *= 127.47). Similarly, for incongruent trials those who received anodal tDCS demonstrated greater reduction in RT (M* *= −246.91, SD* *= 158.87) compared to those in the sham condition (M* *= −72.94, SD* *= 176.34).^3^ See Figure1A for all mean RTs.

### Errors

Error scores were entered into the analysis as raw scores. To examine the impact of tDCS group on error scores on neutral and incongruent trials, an repeated measures ANOVA with tDCS condition (anodal, sham) as the between-groups factor and time (pre-tDCS, post-tDCS) and trial type (neutral, incongruent) as within-groups factors was conducted. There was no statistically significant main effect of tDCS condition on error scores, *F*_1,26_ = 0.05, *P *> 0.001, partial *η*^2^ = 0.002. There was a statistically significant main effect of time, *F*_1,26_ = 12.05, *P *< 0.001, partial *η*^2^ = 0.32, and trial type, *F*_1,26_ = 27.52, *P *< 0.001, partial *η*^2^ = 0.51, on error scores. There was also a statistically significant time × trial type interaction effect, *F*_1,26_ = 4.73, *P *< 0.05, partial *η*^2^ = 0.15. Bonferroni-adjusted pairwise comparisons of error scores revealed a statistically significant difference between pre-tDCS and post-tDCS error scores for incongruent trials, *t*_27_ = 3.46, *P *< 0.05, *d *= 0.83, but no statistically significant difference between pre-tDCS and post-tDCS error scores for neutral trials, *t*_27_ = 1.90, *P *= 0.68, *d *= 0.51. Regardless of tDCS condition (anodal, sham), participants exhibited improved accuracy (decreased error) for incongruent trials only. Participants demonstrated significantly reduced error scores from pre-tDCS (M* *= 3.36, SD* *= 2.57) to post-tDCS (M* *= 1.61, SD* *= 1.57) for incongruent trials.

## Discussion

This study examined the impact of anodal tDCS over the left dorso-lateral prefrontal cortex (DLPFC) on inhibitory control as indicated by performance on a modified Stroop color-word matching task. We predicted that anodal tDCS over the left DLPFC would lead to enhanced performance on the Stroop task relative to sham tDCS. Results revealed that mean Stroop reaction times (RTs) for both neutral and incongruent items were statistically significantly reduced, compared to sham, following anodal stimulation over the left DLPFC. Error rates also decreased although there was no statistically significant effect for tDCS condition. This indicates that the reduced RT did not lead to an increase in errors (i.e., a speed–accuracy trade-off). Participants in the anodal tDCS condition had statistically significant reductions in RT *and* error rates simultaneously relative to the sham condition. Overall, findings provide initial evidence that excitation of the left DLPFC and inhibition of the right DLPFC leads to improvements on an inhibitory control task.

Current findings have important ramifications for the increasing evidence linking inhibitory control to regulation of behavior and adaptive outcomes (e.g., Guerrieri et al. 2008; Hagger et al. 2013a; Guerrieri et al. 2012 #6429; Houben et al. 2011). Our methods were specifically designed to isolate effects of stimulating regions of the brain, specifically, the DLPFC, that have been linked to effective inhibitory control, on response inhibition (Vanderhasselt et al. 2006; Figner et al. 2010; Allom et al. 2015). The Stroop task is acknowledged as a key paradigm to assess response inhibition and, as response inhibition is a fundamental component of the self-control construct (Hofmann et al. 2012), we speculate that current results may have wider implications for regulatory behaviors which require good inhibitory control. For example, research has indicated that reduced inhibitory control is closely associated with behaviors contingent with poorer self-control and reduced behavioral regulation, such as eating and alcohol consumption while, in contrast, better response inhibition capacity is associated with effective self-control and adaptive outcomes (Muraven and Shmueli 2006; Guerrieri et al. 2008, 2012; Houben et al. 2011; Hagger et al. 2013a,b). Furthermore, self-control theorists (Heatherton 2011; Heatherton and Wagner 2011; Harvey 2012; Hagger and Chatzisarantis 2013; Kurzban et al. 2013) and researchers examining individuals performance on response inhibition tasks using imaging techniques (Hedgcock et al. 2012; Friese et al. 2013) have implicated reduced activity in the DLPFC, the region of the brain correlated with motivation and executive functioning, as the mechanism underpinning inhibition failures. This study adds to this body of research by demonstrating that stimulating the same region leads to improved response inhibition on the Stroop task, a task that has been shown to place considerable demand on response inhibition capacity (Hofmann et al. 2012). Current evidence not only provides some corroboration of imaging data but also indicates that stimulating the region leads to adaptive changes in performance on a response inhibition task.

The present findings are also important as they may help to augment the accumulating evidence of research demonstrating that inhibitory control can be improved through engaging in tasks that stimulate response inhibition (Houben et al. 2011; Jones et al. 2011; Rebar et al. 2015; Veling et al. 2011, 2013a,b; Todd and Mullan 2013). These studies demonstrate that engaging in tasks that stimulate inhibitory control over a period of time leads to better behavioral regulation. Researchers surmise that the improvements are due to stimulation of activity in the DLPFC. Our results provide an analog to these findings by demonstrating that stimulating the same region leads to identical effects on response inhibition capacity. The current findings may contribute to the converging evidence for the mechanisms that underpin inhibitory control and an indication of how it can be modulated.

The present findings may have implications for behavioral domains in which lapses in inhibitory control contribute to maladaptive behavioral patterns and associated outcomes. For example, there is evidence that inhibitory control is implicated in eating behavior and conditions associated with overeating such as overweight and obesity (Vohs and Heatherton 2000; Nederkoorn et al. 2010; Hagger et al. 2013b). Reduced left DLPFC activation is associated with both decreased inhibitory control and higher levels of impulsivity in obese populations (Brooks et al. 2013). The finding that increasing activation of left DLPFC improves inhibitory control is therefore consistent with the neurobiological findings concerning brain activation in obesity research, and lends some support to tDCS as a therapeutic intervention in the management of impulsive disorders. Although this study cannot delineate the exact processes underlying improved inhibitory control following excitation of the left DLPFC it could be suggested that anodal tDCS increases synaptic strength and amplifies neural communication in the left DLPFC (Lüscher and Malenka 2012). Alternatively, improved inhibitory control may be attributable to changes in the interhemispheric balance of activation across the DLPFC. The tDCS montage used in this study involved excitation of the left DLPFC and inhibition of the right DLPFC, which would lead to asymmetric interhemispheric activation. It cannot therefore be concluded that increased activation of left DLPFC alone led to improved inhibitory control. Fecteau et al. (2007) suggested that altering the interhemispheric balance of DLPFC activation impacts upon risk taking, which may be the case in this study.

The present findings are analogous to improvements in executive functioning, specifically planning ability following activation of the left DLPFC (Dockery et al. 2009; Leite et al. 2012). As for tasks invoking planning skills, only increased activation of the left DLPFC has been associated with improved inhibitory control in this study. Direct current stimulation of the DLPFC has been shown to reduce alcohol cravings, a behavior thought to be indicative of resilience. For example, researchers have reported that the contralateral application of tDCS decreased cravings for people with alcohol dependence (Boggio et al. 2008) and less risk-taking behavior in marijuana users (Boggio et al. 2010), regardless of the left-right configuration of the anodal-cathodal electrodes over the DLPFC. Similarly, Fregni et al. (2008) reported that smokers experienced a reduced number of nicotine cravings after cross-hemispheric tDCS over both the left and right DLPFC. Boggio et al. (2008) suggested that anodal stimulation or cathodal inhibition of the right or left DLPFC disrupted the balance between the left and right DLPFC activation, such that cravings were reduced. The present findings, however, suggest that increased excitation of the left DLPFC specifically improves inhibitory control. It may be suggested that the pattern of DLPFC involvement depends upon the nature of the self-control ‘task’. There is some evidence to suggest that neuronal activation is much more bilaterally distributed across the DLPFC during craving states (Wilson et al. 2004). We therefore suggest that the left DLPFC is primarily activated when response inhibition, or more basic inhibitory control, is required.

Some limitations of this study should be considered. The most important limitation is that we cannot determine whether improved inhibitory control resulted from increased excitation of left DLPFC, or from changing the balance of activity across both DLPFC cortices. In addition, we cannot confirm that tDCS did not impact on other densely connected areas of prefrontal cortex, such as the ventromedial prefrontal cortex, an area that has also been implicated in self-control (Hare et al. 2009). In addition, we did not collect data examining the effect of TDCS conditions on responses to a simple choice-time reaction task. Comparison of such data with current findings would have tested whether the effects of stimulation of the DLPFC were confined to tasks tapping response inhibition rather than a generalized enhancement of cognitive functioning as the improvements in the neutral Stroop items found in the current study might imply. Collection of data on tasks tapping generalized reaction time as a comparison condition would be necessary to draw unequivocal conclusions as to the nature of the effect. Finally, we did not assess a behavioral manifestation of self-control beyond that of response inhibition using the Stroop. It would be informative to examine the impact of changes in response inhibition on other behaviors requiring or invoking inhibitory control such as caloric intake or alcohol consumption.

This study demonstrates improvements in inhibitory control in people receiving tDCS and is a step toward understanding the neural underpinnings of self-control. The implication is that tDCS may be a valuable strategy for the prevention or treatment of problems relating to self-control. Deficits in response inhibition have also been demonstrated to contribute to clinically-diagnosed impulse disorders, such as obsessive compulsive disorder and attention deficit hyperactivity disorder (Chamberlain and Sahakian 2007). The potential of tDCS as a therapeutic tool for such disorders also remains unexplored. Future tDCS studies should investigate the optimum dose, duration, and electrode configuration required to strengthen the self-control process in both nonclinical groups and those with clinically diagnosed impulse disorders.

## Footnotes

1 It is important to note that incongruent or ‘conflict’ versions of the Stroop task, in which individuals are presented with Stroop items that create interference between color-naming and word-reading processes and forcing a suppression of the prepotent response to read the word, taps response inhibition. The Stroop task has been widely acknowledged as one of the predominant paradigms to evaluate response inhibition (Logan et al. 1997; Hofmann et al. 2012) and it has given rise to other tasks that tap the same inhibition processes (Bush et al. 2003). Furthermore, authors have consistently linked inhibitory control, self-control resource depletion, and Stroop task performance (Hagger et al. 2010; Hofmann et al. 2012). Although response inhibition appears to be the focal process underpinning Stroop performance, it is important to recognize that the task is also implicated in other aspects of cognitive processing and executive functioning including selective attention, detection interference, task switching, and cognitive flexibility (Roberts and Hall 2008; Hyafil et al. 2009; Xiao et al. 2009; Coderre and van Heuven 2013).

2 The version of the Stroop task used in the current experiment is a variant of the single-item computerized version (MacLeod et al. 2005) and taps equivalent word-reading and color-naming processes and the associated interference. The version adopts a protocol first proposed by Dyer (1973) to test the effect of separating the reading and color processes in the presentation of the stimuli on the Stroop effect rather than versions where the reading and color-naming processes in the stimuli are integrated (words appearing in different colored ink), which are more ‘traditional’, as noted by MacLeod (1991; MacLeod et al. 2005). So comparing reaction times to neutral stimuli (e.g., where the string of letters appearing at the bottom of the screen in gray reads “BLUE”, and the string of letters appearing at the top reads “NGSL” and appears in the color blue) with reaction times for incongruent stimuli (e.g., string of letters appearing at the bottom of the screen in gray reads “BLUE”, and the string of letters appearing at the top is a color word “RED”, and appears in the color blue). Dyer tested a number of variants of these conditions to examine the effect of separation of reading and color-naming processes, and his data showed that the Stroop effect tended to be slightly weaker in the separate conditions compared to the integrated conditions, but the effect was still clear. Of relevance to the current study, Dyer's data clearly demonstrated the Stroop effect for sets of stimuli equivalent to those used in the current version of the Stroop task. Specifically, he found that reaction times for the congruent stimuli (termed “incongruent different A”) were significantly slower than the neutral stimuli (termed “control same”), consistent with the Stroop effect. Based on this developmental work with the Stroop task, we are confident that the ‘separated’ version used in the current research taps equivalent processes to versions in which the stimuli are integrated.

3 For completion, we also conducted these analyses without using change scores and using pre-tDCS Stroop RT scores as covariates. Specifically, in this alternative set of analyses, we compared the effect of tDCS condition (anodal, sham) on post-tDCS Stroop RT scores for neutral and incongruent trials separately while controlling for pre-tDCS scores from both trial types. A one-way ANCOVA with post-tDCS Stroop RT for neutral trials as the dependent variable, tDCS condition as the independent variable, and pre-tDCS Stroop RT scores for neutral and incongruent trials as covariates revealed that participants receiving ALCR tDCS exhibited statistically significantly faster Stroop RT scores (adjusted M = 750.043, SE = 33.218) relative to participants receiving sham tDCS (adjusted M = 880.87, SE = 33.22), *F*_1,24_ = 7.51, *P *= 0.011, partial *η*^2^ = 0.24. An identical ANCOVA, with post-tDCS Stroop RT for incongruent trials as the dependent variable, tDCS condition as the independent variable, and pre-tDCS Stroop RT scores for neutral and incongruent trials as covariates revealed that participants allocated to the ALCR tDCS condition exhibited statistically significantly faster Stroop RT scores (adjusted M = 908.48, SE = 46.53) relative to participants allocated to the sham tDCS condition (adjusted M = 1072.41, SE = 46.53), *F*_1,24_ = 6.01, *P *= 0.022, partial *η*^2^ = 0.20. Results mirror the pattern of effects using change scores.
